# The untapped potential of vaginal microbiome diagnostics for improving women’s health

**DOI:** 10.3389/fcimb.2025.1595182

**Published:** 2025-08-07

**Authors:** Ana Sofia Da Silva, Saba Anwar, Suenie Park, Sunwha Park, Laura Goodfellow, Chrysi Sergaki

**Affiliations:** ^1^ Department of Urogynaecology, King’s College Hospital, London, United Kingdom; ^2^ Science and Research, Medicines and Healthcare Products Regulatory Agency, Potter’s Bar, United Kingdom; ^3^ Microbiome Research Center, BiowaveW, Corelab, Seoul, Republic of Korea; ^4^ Department of Obstetrics and Gynaecology, College of Medicine, Ewha Medical Research Institute, Ewha Womans University, Seoul, Republic of Korea; ^5^ Women’s and Children’s Health, University of Liverpool, Liverpool, United Kingdom

**Keywords:** vaginal microbiome, diagnostics, standardisation, women’s health, personalised treatment

## Abstract

Microbiome research has expanded rapidly over the past 15 years; however, the primary focus has been on the gut microbiome. Although understudied, the vaginal microbiome holds significant potential to improve women’s health. In this paper, we describe the current clinical diagnostic techniques utilised in women’s health and examine their drawbacks and limitations. We also discuss emerging diagnostic technologies based on microbiome analysis that could enable greater precision in diagnosis and personalised treatment. We additionally emphasise the need for standardisation in microbiome analysis and strengthening the knowledge base to enable advancements in accurate diagnosis, ultimately improving patient outcomes. This article aims to highlight opportunities in the field that can transform women’s health outcomes and outline the necessary actions to realise these opportunities, thereby enhancing women’s lives.

## 1 Introduction

The vaginal microbiome is a complex and dynamic ecosystem of microorganisms that plays a crucial role in maintaining vaginal health. Its composition is influenced by hormonal fluctuations occurring during different stages of a woman’s life, such as prepuberty, the reproductive years, and postmenopause (Hickey et al., 2012; Chen et al., 2021). During the reproductive years, cyclical hormonal fluctuations associated with the menstrual cycle drive shifts in the vaginal microbiome (Hickey et al., 2012). The vaginal microbiome’s ability to adapt and return to a state of homeostasis is essential for protecting against infections and maintaining overall vaginal health (Chee et al., 2020). Disruption of this delicate balance can lead to dysbiosis, which may adversely affect reproductive health and contribute to conditions such as infertility, miscarriage, gynaecological cancers, premature birth, and disease (Merone et al., 2022).

Despite advances in research, our understanding of the vaginal microbiome and its interactions with the host remains limited. Historically, medical studies have excluded female participants and research data have been collected from men and subsequently generalised to women (Merone et al., 2022). Geller et al. analysed 86 randomised controlled trials across nine journals and found female representation to be just 37% (Geller et al., 2011). The underrepresentation of women in clinical trials limits understanding of gender differences in drug treatments, potentially leading to harmful outcomes. This bias can result in less effective treatments for women and overlooked side effects due to factors such as hormonal fluctuations, pregnancy, and menopause. Without adequate representation, safety profiles may be misunderstood, and women may not receive the correct diagnosis or treatment. A report conducted by the Global Health Alliances in 2014 suggested that neglect in medical research and funding is directly responsible for delayed diagnosis, severe disease progression, and premature death in women (It’s about time to focus on women’s health, 2023). Fewer studies focusing on women’s health issues will lead to slower development of diagnostics and treatments specifically designed to address women’s specific health needs. This can hinder progress in areas such as reproductive health, menopause, and certain cancers, including breast, cervical and ovarian cancer. Numerous microbiome studies highlight the need for personalised healthcare approaches that account for individual variations (Kashyap et al., 2017; Ratiner et al., 2024). Vaginal microbiome diagnostics show promise in various applications, including early detection of infections, more accurate diagnosis of gynaecological conditions, and improved reproductive health outcomes by assessing fertility, pregnancy, and cancer risks. Personalised treatment plans can also be tailored based on these diagnostics. In this review, we evaluate the potential of different rapid diagnostic tools, current advancements in vaginal microbiome diagnostics, and their prospects.

## 2 Vaginal microbiome diagnostics for vaginal infections

The dominant bacteria in the vaginal microbiome are *Lactobacillus* species, which produce lactic acid, helping to maintain a healthy acidic pH ranging from approximately 3.5 to 4.5 (Ravel et al., 2011). This acidic environment is essential for inhibiting the growth of pathogenic organisms and supporting overall vaginal health (Witkin and Linhares, 2017). In contrast, higher, more alkaline pH levels are associated with disruptions in microbial balance and an increased risk of vaginal infections (Chee et al., 2020). Alterations in vaginal pH, often resulting from decreased lactate production, are linked to vaginal dysbiosis and have been observed in patients with conditions such as bacterial vaginosis, chlamydia, and vulvovaginal candidiasis (Ceccarani et al., 2019).

### 2.1 Bacterial vaginosis

Bacterial vaginosis (BV) is the most common form of vaginitis in women of reproductive age, with a global prevalence ranging from 23% to 29% (Bacterial vaginosis; Peebles et al., 2019). It is characterised by a reduction in lactic acid-producing *Lactobacillus* species and an overgrowth of other bacterial species, such as *Gardnerella vaginalis*, *Atopobium vaginae*, and various anaerobes (Eschenbach, 1993; Srinivasan et al., 2012; Muzny et al., 2018). This shift in microbial composition leads to an increased vaginal pH, creating a more alkaline environment that promotes the overgrowth of opportunistic pathogens (Witkin and Linhares, 2017). Current diagnostic methods for BV include clinical approaches such as the Amsel criteria and Nugent score (Amsel et al., 1983; Nugent et al., 1991), as well as molecular tests like PCR that detect specific bacterial DNA (Cartwright et al., 2012). Future advancements in BV diagnostics, particularly the integration of technologies like Next-Generation Sequencing (NGS), will enable more precise identification of specific bacterial species and strains, along with their relative abundances (Srinivasan et al., 2012; Yeoman et al., 2013; Wang et al., 2015; Ferreira et al., 2018). In practice, a vaginal swab specimen would be collected and subjected to DNA extraction followed by NGS to profile the vaginal microbiota. The resulting microbial composition would be analysed to detect the presence and relative abundance of bacterial species associated with BV. This enhanced level of detailed characterisation of the microbes involved will enable clinicians to distinguish between different classifications of dysbiosis and guide targeted treatment approaches, which will not only improve patient outcomes but also contribute to antimicrobial resistance (AMR) stewardship. The value of clinical metagenomics for bacterial infection diagnosis has been extensively discussed (d’Humières et al., 2021; Hassall et al., 2024); however, its potential for BV diagnosis is often overlooked.

### 2.2 Sexually transmitted infections

The World Health Organisation (WHO) estimated that in 2020, there were 374 million new cases of sexually transmitted infections (STIs) globally among individuals aged 15 to 49. This included 129 million cases of chlamydia, 82 million cases of gonorrhoea, 7.1 million cases of syphilis, and 156 million cases of trichomoniasis (Sexually transmitted infections (STIs)). While many STIs are treatable with a short course of antibiotics, they pose significant health risks for women, including infertility, cervical cancer, preterm labour, and pelvic inflammatory disease (McCormack and Koons, 2019). Human papillomavirus (HPV) is of particular concern, as persistent infections with high-risk HPV types are the leading cause of cervical, vulval, and vaginal cancers (Okunade, 2020). Numerous studies have shown that disruption of the vaginal microbiota, particularly a low abundance of *Lactobacillus* species, is associated with an increased incidence of STIs (Peipert et al., 2008; Van De Wijgert et al., 2009; Gosmann et al., 2017; McClelland et al., 2018). Specific alterations in the vaginal microbiome may serve as early biomarkers for detecting vaginal infections, potentially before symptoms manifest. In practice, a vaginal swab specimen would be collected and processed for DNA extraction followed by NGS to characterise the microbial community. The relative abundance of *Lactobacillus* species will be compared to that of bacterial species associated with dysbiosis, enabling assessment of microbiome composition and its potential link to increased susceptibility to STIs. Early detection could play a central role in preventing adverse outcomes of STIs, such as pelvic inflammatory disease, infertility, and cancers (Peipert et al., 2008; Van De Wijgert et al., 2009; Gosmann et al., 2017; McClelland et al., 2018). In addition, regular assessments of the vaginal microbiome can help monitor treatment effectiveness and manage recurrent infections by tracking microbial diversity and guiding timely interventions. While metagenomic approaches hold promise for becoming part of routine clinical STI diagnosis, several additional factors must be addressed. These include developing suitable laboratory protocols and standards to prevent contamination and ensure accurate result interpretation, reducing the cost and turnaround time of instrumentation, and establishing effective bioinformatic analysis pipelines (Caruso et al., 2021).

Incorporating microbiome analysis into vaginal infection diagnostics could offer several advantages, including improved pathogen detection, enhanced understanding of disease progression, and the potential to develop personalised treatment plans.

## 3 Vaginal microbiome diagnostics in gynaecology oncology

Gynaecological cancers account for 14.4% of new cancer diagnoses in women globally (Sung et al., 2021), with cervical cancer being the most prevalent and responsible for approximately 350,000 deaths in 2022 (Cervical cancer). This malignancy is primarily caused by persistent infection with high-risk HPV (Yim and Park, 2005), which is more likely in women with dysbiosis of the vaginal microbiota. This is thought to occur through two mechanisms: first, an overgrowth of microbes that characterise dysbiosis, such as *Gardnerella vaginalis*, which can promote chronic inflammation and increase the risk of DNA damage (Han et al., 2021). Second, a reduced abundance of *Lactobacillus* leads to a higher vaginal pH, making the environment more susceptible to HPV infections (Wang et al., 2023). In contrast, women exhibiting a vaginal microbiota characterised by low diversity and a high relative abundance of *Lactobacillus* species tend to show reduced susceptibility to HPV infection. Therefore, women with a diverse, *Lactobacillus*-depleted microbiome are at a greater risk for cervical intraepithelial neoplasia (CIN) and the development of cervical cancer (Mitra et al., 2015; Brusselaers et al., 2019; Wei et al., 2022). Conversely, women with a vaginal microbiome dominated by *Lactobacillus* species are more likely to experience natural regression of CIN without treatment (Mitra et al., 2020).

Arguably the incorporation of HPV testing into cervical screening programmes has been an early example of the role of vaginal microbiota diagnostics in clinical healthcare. To advance this field, recent efforts have focused on employing artificial intelligence (AI) to analyse the vaginal microbiota of women with and without cervical cancer, aiming to reveal the microbial mechanisms involved in cancer development (Sekaran et al., 2023). Understanding these interactions could provide deeper insights into cancer pathogenesis and thereby improve treatment options (Sharifian et al., 2023). Furthermore, integrating self-administered or point-of-care vaginal microbiota diagnostics into cervical screening programmes could offer a more patient-friendly alternative to traditional screening methods. This approach may aid in triaging women for further assessment by healthcare providers and enable timely interventions for dysbiosis, ultimately improving overall cervical cancer health outcomes (Casas et al., 2022).

Emerging research suggests a potential link between the microbiome and endometrial cancer, with persistent inflammation leading to increased activation of inflammatory cytokines and subsequent tumorigenesis at the endometrial level (Aquino et al., 2024). However, the evidence remains limited, and further research is required. Some studies indicate that women with endometrial cancer have a distinct vaginal and endometrial microbiome composition compared to healthy women. For example, an increased abundance of *Micrococcus* has been associated with endometrial cancer and various other cancers, highlighting this genus as a potential diagnostic target (Richard et al., 2018). In principle, point-of-care diagnostic testing could be developed using *Micrococcus* as a biomarker, where an increased abundance of this genus in endometrial biopsy samples may serve as an early indicator of endometrial cancer or other malignancies. Current diagnostic methods for endometrial cancer include imaging to detect thickening of the endometrial lining, followed by invasive endometrial sampling. The advent of less invasive diagnostic techniques, such as vaginal microbiome analysis for comprehensive profiling of the microbial communities, could improve the ease and acceptability of diagnostic testing. This approach has the potential to reduce the number of invasive procedures, enhance early cancer detection, and improve patient adherence. Ovarian cancer is the eighth most common cancer among women worldwide and the fourth leading cause of cancer-related deaths in women (Ovarian cancer statistics). Due to nonspecific symptoms, ovarian cancer is often diagnosed at advanced stages, leading to a poorer prognosis. Currently, biomarker cancer antigen 125 is used as a diagnostic tool; however, it lacks specificity, as elevated levels can occur in various conditions such as endometriosis, liver disease, leiomyoma, and pelvic inflammatory disease (The CA125 blood test for ovarian cancer has been re-evaluated). This underscores the need for novel tools for ovarian cancer diagnostics. Although evidence linking altered microbiomes with ovarian cancer is limited, some studies have shown associations between ovarian cancer and elevated levels of *Prevotella*, *Bacteroides*, and *Proteobacteria*, alongside reduced levels of *Ruminococcus* and *Actinobacteria (* Jacobson et al., 2021; Choi and Choi, 2024). Notably, *Prevotella* has also been associated with endometrial and cervical cancer and is linked to a proinflammatory state (Ley, 2016; Li et al., 2021). Given its noninvasive nature, exploring the microbiome as a diagnostic tool offers valuable benefits, including higher patient adherence, lower risk of complications, quicker recovery, increased accessibility, and broader diagnostic reach. However, further research is required to strengthen our knowledge base.

## 4 Vaginal microbiome diagnostics for polycystic ovary syndrome

Polycystic ovary syndrome (PCOS) is an endocrine disorder affecting up to 20% of reproductive-aged women worldwide and is one of the most prevalent gynaecological disorders. While the pathophysiology of this syndrome has been described, the cause is not well understood; however, recent evidence has associated microbial composition with PCOS. It has been hypothesised that, due to the endocrine cause of PCOS and the involvement of microbes in hormonal regulation, microorganisms may play a potential role in PCOS (Geller et al., 2011). Current literature indicates a greater abundance of particular microbes in PCOS patients compared to healthy controls, including *Mycoplasma*, *Prevotella*, *Actinomyces*, *Gardnerella*, and *Streptococcus* species (Sola-Leyva et al., 2023). One study reported that *Mycoplasma* was the most distinguished genus in PCOS, and the women with a relative abundance of more than 0.02% of *Mycoplasma* in the vaginal microbiome were at high likelihood of having PCOS, indicating its potential as a biomarker for PCOS screening (Hong et al., 2020). Similarly, *Actinomyces* species were found to be significantly more abundant in non-*Lactobacillus*-dominated PCOS patients in comparison to *Lactobacillus*-dominated healthy patients and have been suggested as a potential biomarker (Pereira et al., 2024). These findings identify potential biomarkers that could be implemented in clinical settings as part of a routine point-of-care test for the diagnosis of PCOS, offering faster results, improved accessibility, and the potential for earlier intervention and personalised treatment in clinical settings. However, additional studies are necessary to validate this association and determine whether the increased abundance of these species is specific to PCOS, thereby supporting their potential use as reliable and clinically significant biomarkers. In addition, it is important to note that there is limited research on this topic, and more well-designed studies are needed. Studies with larger sample sizes, appropriate negative and positive controls, control for key confounders, and proper case–control matching would be crucial (Khan et al., 2010; Ser et al., 2023).

## 5 Vaginal microbiome diagnostics for endometriosis and adenomyosis

### 5.1 Endometriosis

Endometriosis is a chronic, oestrogen-dependent gynaecological condition affecting approximately 10% of reproductive-age women (Wessels et al., 2021). Currently, the gold standard for diagnosis is laparoscopy. Researchers have found a bidirectional relationship between the microbiome and the development of endometriosis, indicating that any changes in the host’s microbiome can significantly affect the onset and progression of the condition. This highlights the critical need to explore the connection between the microbiome and endometriosis to allow earlier, more accurate diagnosis and the potential for personalised treatment strategies based on individual microbiome profiles (Ser et al., 2023). A study conducted by Ata et al., sampling 14 women with stage 3/4 endometriosis and 14 healthy controls, noted the complete absence of *Gemella* and *Atopobium* species in the endometriosis group (Ata et al., 2019). However, due to the small sample size, further studies must be conducted with larger sample sizes to confirm this finding. Further investigation is needed to better understand the condition and its link to the vaginal microbiome.

### 5.2 Adenomyosis

Adenomyosis is a benign gynaecological condition commonly found in women of reproductive age, with unclear aetiology. Pathological diagnosis after surgery is the most accurate diagnostic tool; however, imaging with ultrasound and MRI can identify features of adenomyosis. Novel, less invasive diagnostic methods, such as microbiome diagnostics, could have a role and may aid in understanding the condition better. A study conducted by Pan et al. (2024) observed differences in the vaginal microbiome between patients with adenomyosis and healthy individuals. At the phylum level, the relative abundance of Firmicutes in the adenomyosis group was higher than in the control group. At the genus level, the relative abundance of *Gardnerella* in the adenomyosis group was significantly lower than that of the control group. In a separate study (Kunaseth et al., 2022), vaginal microbiota richness was observed to be significantly higher in the adenomyosis group, with differences in bacterial abundance observed for both groups. *Megaspehera*, *Fastidiosipila*, Hungateiclostrsidiaceae, and *Clostridia* were more frequently found in the vaginal microbiota in the control group. Whereas *Alloscardovia*, Oscillospirales, Ruminococcaceae, *UCG_002*, *Oscillospiraceae*, *Enhydrobacter*, *Megamonas*, Moraxellaceae, *Subdoligranulum*, Selenomonadaceae, and *Faecalibacterium* in the vaginal microbiota were significantly higher in the adenomyosis group compared to the controls. The observed differences in bacterial abundance between the control and adenomyosis groups highlight the potential of specific microbial species to serve as biomarkers for the condition. Vaginal swab samples can be noninvasively used for DNA extraction and sequencing to detect bacterial signatures associated with adenomyosis, assess an individual’s risk of having adenomyosis, and possibly limit their need for surgery. Further research into the vaginal microbiome and adenomyosis will enhance our understanding of the disease and offer valuable insights for the development of noninvasive diagnostic methods and personalised, precision treatments.

## 6 Vaginal microbiome diagnostics for fertility

Infertility is clinically defined as the inability to conceive after 12 months of appropriately timed, regular, unprotected sexual intercourse (Khan et al., 2010). In 2023, the WHO estimated that infertility affects approximately 12.6% of the adult population at any given time (World Health Organization, 2021). Recent research has increasingly focused on the role of the female reproductive tract microbiota, particularly the vaginal microbiome, in both baseline fertility and fertility treatment outcomes (Koedooder et al., 2019; Gao et al., 2022; Vitale et al., 2022). This growing interest has spurred the development of commercial microbiota testing services marketed to predict fertility outcomes (EMMA; Endometrial Health; Invivo Healthcare), based on evidence that a more diverse vaginal microbiota and a depletion of *Lactobacillus* species are linked to reduced baseline fertility (Vitale et al., 2022) and lower success rates in assisted reproductive technologies such as *in vitro* fertilisation (IVF) (Koedooder et al., 2019). However, efforts to modify an “unfavourable” vaginal microbiota profile during fertility treatment have not consistently resulted in sustained changes (Invivo Healthcare; Budding et al., 2010; Jepsen et al., 2022). Consequently, a 2020 expert consensus from European fertility specialists recommends against routine microbiota testing in asymptomatic women, citing insufficient clinical evidence (García-Velasco et al., 2020). While the knowledge base requires further strengthening, emerging research suggests that monitoring the vaginal microbiome may offer benefits in fertility treatment. Tracking microbiome composition could help healthcare providers identify optimal treatment windows, as the vaginal microbiota is constantly shifting and can naturally move toward a more favourable profile (Budding et al., 2010; Jepsen et al., 2022). Additionally, microbiome assessments might detect subclinical dysbiosis that impacts fertility outcomes, further supporting the development of more personalised treatment plans. Although further clinical trials are needed, microbiome diagnostics hold promise for enhancing fertility treatment strategies through noninvasive, targeted approaches.

## 7 Vaginal microbiome diagnostics for pregnancy-related complications

Improving maternity care has garnered considerable political interest. In 2015, the UK National Maternity Safety Ambition set a goal to halve the rates of stillbirth, neonatal and maternal death, and brain injuries associated with birth by 2025 (Department of Health, 2016). The association between the vaginal microbiota and preterm birth became an early focus of NGS, as approximately 40% of preterm births are believed to be related to infection, with the anatomical proximity of the vagina to the uterine cavity making it a plausible source of infection (Lamont and Sawant, 2005). In this context, “infection” may involve organisms that are typically commensal in nonpregnant women. Notably, women with “low lactobacilli” vaginal microbiota are considered at higher risk of delivering preterm (Gudnadottir et al., 2022; Huang et al., 2023). Interestingly, the vaginal microbiota is a better predictor of early than late preterm birth; this is concordant with historical work showing the infection is implicated more often in early than late preterm birth (Lamont and Sawant, 2005). This has led to interest in developing a clinically applicable near-patient test for the vaginal microbiota of pregnant women at risk of preterm birth. Direct on-swab metabolic profiling by Desorption Electrospray Ionisation Mass Spectrometry (DESI-MS) is a technique that uses specific metabolome signatures to simultaneously predict both the composition of the vaginal microbiome and host inflammatory status (Pruski et al., 2021). It is hoped that, in the future, such a device may be able to identify women at risk of preterm birth with an abnormal microbiota that is amenable to modification, and which could, in turn, increase their chance of a term birth (Bayar et al., 2023).

Women with preterm prelabour rupture of membranes (PPROM) may benefit from well-developed vaginal microbiota diagnostics (Bennett et al., 2020). Ruptured membranes increase susceptibility to ascending infections from the vagina, which can lead to neonatal sepsis and maternal chorioamnionitis. Delaying birth after membrane rupture, particularly before 34 weeks, improves neonatal outcomes by allowing greater foetal maturity. Women with PPROM fall into two groups: those with a lactobacilli-dominant vaginal microbiota before and shortly after PPROM, and those with pre-existing dysbiosis before PPROM (Brown et al., 2019). Prophylactic antibiotics are typically administered after PPROM (erythromycin in UK practice) (Thomson, 2019). In a small observational study, women with PPROM and a lactobacilli-dominant microbiota who received erythromycin experienced alterations in their microbiota, notably depletion of lactobacilli. This depletion was associated with early-onset neonatal sepsis (Brown et al., 2018). Conversely, women with lactobacilli-depleted microbiota of PPROM may have benefited from erythromycin, as it reduced microbial richness and diversity, which was linked to a lower risk of chorioamnionitis. Future use of vaginal microbiota diagnostics could support the characterisation of the vaginal microbiota at the time of PPROM to guide whether prophylactic antibiotics are administered, or not, and thereby either maintain a lactobacilli dominant vaginal microbiota, or alter lactobacilli-depleted microbiota, and in turn reduce chorioamnionitis and neonatal sepsis.

A reduction in vaginal lactobacilli species has been observed in women who later experience first-trimester miscarriage (Al-Memar et al., 2020; Shahid et al., 2022), suggesting that vaginal microbiota diagnostics could help identify a modifiable risk factor. In contrast, associations between vaginal microbiota and other major obstetric syndromes, such as preeclampsia, growth restriction, and stillbirth, are less well established, with research on the role of microbiota diagnostics in these conditions still in its infancy (Ishimwe, 2021; Baud et al., 2023; Holliday et al., 2023). While further research is required, these observations suggest that vaginal microbiome analysis could serve as a valuable adjunct to enhance pregnancy outcomes by identifying at-risk individuals and offering targeted treatments. Furthermore, integrating microbiome profiles into existing pregnancy risk prediction models could prove to be a beneficial addition (Saadaoui et al., 2023). This approach may facilitate timely interventions and preventive strategies, ultimately contributing to improved maternal and foetal health outcomes (Peelen et al., 2019).

## 8 Vaginal microbiome diagnostics and genitourinary syndrome of menopause

At this time, women will be spending 40% of their lives in the postmenopausal state, with 50%–70% of postmenopausal women reporting symptomatic genitourinary syndrome of menopause (GSM) (Da Silva et al., 2021). GSM is the accepted term used to describe the broad range of signs and symptoms affecting the genitourinary tract, which occur due to the loss of endogenous sex steroids associated with menopause (Portman et al., 2014). It has been observed that, in postmenopausal women, there is a decrease in *Lactobacillus* species and a higher abundance of genera including *Anaerococcus*, *Peptoniphilus*, and *Prevotella*, which could play a role in the clinical presentation of GSM (Gliniewicz et al., 2019). Consequently, a microbiome-based diagnostic test that monitors the abundance of *Anaerococcus*, *Peptoniphilus*, and *Prevotella* species may offer valuable insights into the pathogenesis and progression of GSM. A *Lactobacillus*-dominant vaginal microbiome is vital in maintaining an acidic pH and protecting against bacterial pathogens (Taithongchai et al., 2024). Postmenopausal women are noted to have high vaginal pH, which results in the loss of local vaginal defences against harmful microorganisms, predisposing them to infections such as urinary tract infections (UTIs) (Harding et al., 2024). The prevalence of UTIs is 20% in women over 65, compared with 11% in the general population (Chu and Lowder, 2018). While more studies are starting to emerge, there remains a lack of knowledge about the composition of the vaginal microbiota across the stages of menopausal transition, its interactions with hormones such as oestrogen, and its associations with infections. Evaluating changes in the vaginal microbiota across a woman’s lifespan can provide valuable insight for healthcare providers to help better understand menopausal wellness and its associated morbidities.

## 9 Enabling implementation of microbiome analysis in diagnostics

While the need for improved understanding of how the vaginal microbiome has an impact on women-specific issues such as infertility or gynaecological cancers, a major hurdle in covering this knowledge gap is the variability in microbiome data across studies, as well as the insufficient research in women’s health. This observed variability results from both the clinical trial design and the methodologies used. Current methods used to study the composition of the microbiome introduce biases at different steps of analysis; therefore, the adoption of vaginal microbiome-specific reference reagents (RRs) is essential to enhance the reproducibility and comparability of research findings across studies worldwide. This effort has been initiated by the Medicines and Healthcare Products Regulatory Agency (MHRA) with the development of the WHO gut microbiome-specific International RRs, while WHO-endorsed vaginal-specific microbiome RRs which will support the development, optimisation, and implementation of vaginal microbiome diagnostics and the harmonisation and reproducibility of clinical studies (Sergaki et al., 2022; Anwar et al., 2023). Furthermore, to progress in the field of vaginal microbiome diagnosis, we need to strengthen the knowledge base on the factors associated with changes to the microbiome and ensure that all relevant variables are appropriately captured in clinical studies. Numerous factors, such as age, hormone status (menstrual cycle, birth control pills, menopause, pregnancy), infections, and sexual behaviours, play a significant role in the variability of the vaginal microbiome and the clinical trial observations (White et al., 2011; Huang et al., 2014). A robust clinical trial design with comprehensive metadata will allow for a deeper understanding of how these factors influence the vaginal microbiome and will help to identify the necessary considerations for ensuring an accurate diagnosis.

## 10 Bringing vaginal microbiome diagnostics into clinical use

The lack of precision in current diagnostic tools for women’s health remains a significant obstacle to timely detection, resulting in delayed diagnoses and suboptimal treatment outcomes. To bridge this gap, a shift is needed to prioritise gender-specific research, refine diagnostic methodologies, and enhance clinician training. The vaginal microbiome represents a promising area in women’s health diagnostics, offering potential biomarkers that could revolutionise early detection and intervention. While further research is needed to strengthen the links between alterations in the vaginal microbiome and specific conditions, as discussed previously, several studies have already demonstrated the clinical value of microbiome-based diagnostics. For example, cervicovaginal fluid (CVF) samples from pregnant women have been analysed to identify bacterial markers predictive of preterm birth, with the potential to develop models that predict preterm birth when integrated with clinical data (Park et al., 2021; Park et al., 2022). The success of HPV vaccination and screening programmes in preventing cervical cancer exemplifies the critical role of pursuing biomarker-driven diagnostics in public health, allowing health concerns to be identified at their earliest stages when treatment can be most effective, and lead to better patient outcomes. To advance this effort, we must address the persistent underrepresentation of women in clinical trials and ensure that ongoing research includes sufficiently large sample sizes. This is crucial for uncovering novel biomarkers and therapeutic targets within the vaginal microbiome, allowing for more effective diagnostic tools and personalised treatments. The incorporation of microbiome diagnostics into clinical workflows represents a critical advancement in women’s health, offering the potential to enhance existing diagnostic tools and support the development of novel POCT strategies. Traditional diagnostic methods, such as Amsel’s criteria or Nugent scoring, commonly used to diagnose BV, are often limited by subjectivity and variability. In contrast, emerging molecular-based assays offer a more accurate and comprehensive analysis of the vaginal microbiota. These approaches provide greater insight into the microbial community, enabling more precise identification of dysbiosis and associated reproductive health risks. To ensure the successful clinical application of microbiome-based diagnostics, rigorous clinical trials must validate microbiome diagnostics and, along with appropriate standardisation, strengthen the knowledge and evidence base for their use in clinical settings, ensuring regulatory compliance of such approaches and optimising their integration into clinical workflows to enhance women’s reproductive health and quality of life.

## 11 Conclusions

**Figure 1:**
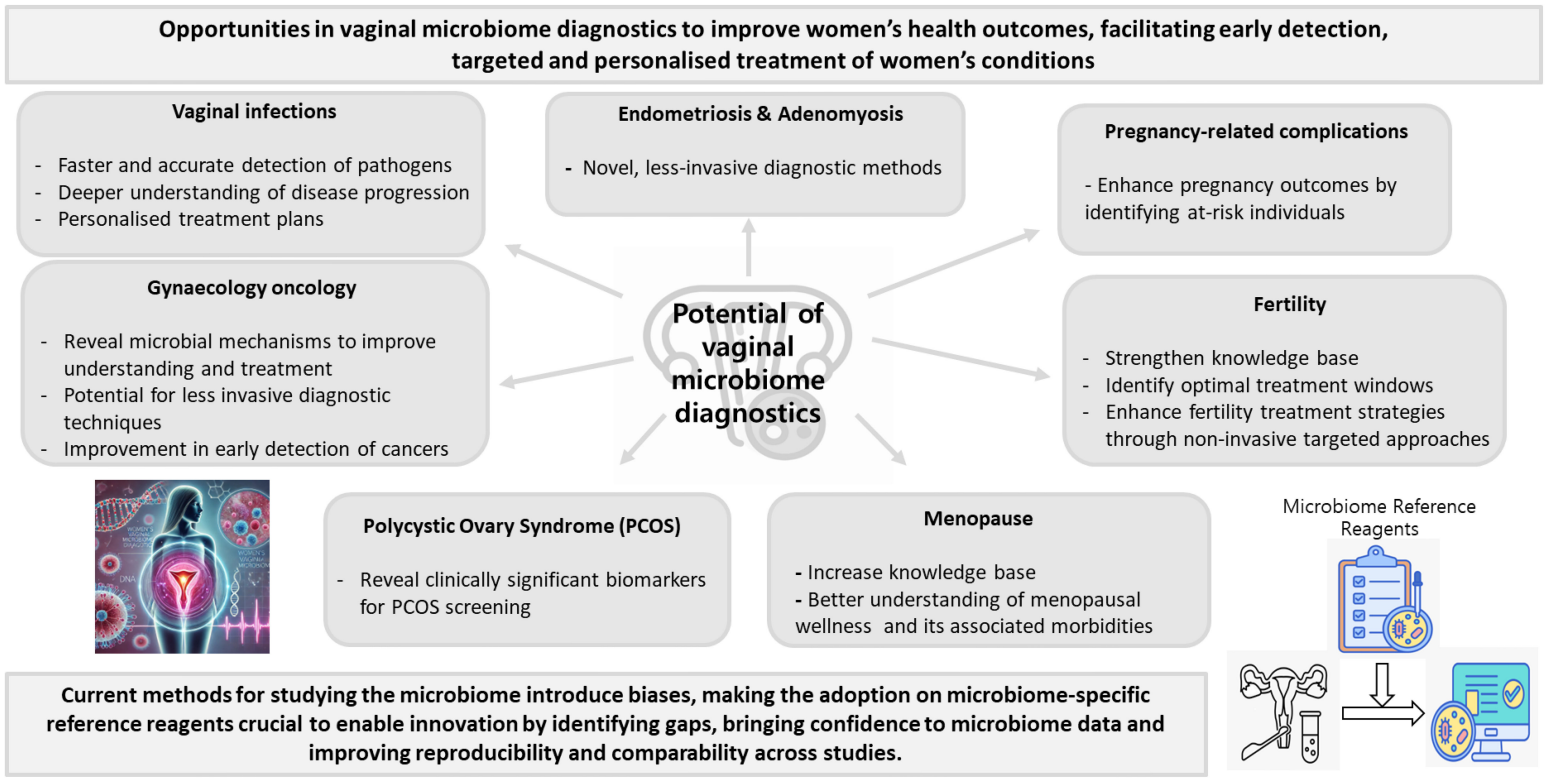
Potential of vaginal microbiome diagnostics in advancing women’s health (The icons on the right were obtained using freely available resources from Freepik, and the image on the left was generated with ChatGPT 4.0).

While women’s health, and particularly the vaginal microbiome, are under-researched, the potential of vaginal microbiome diagnostics in improving women’s health is substantial, offering a promising avenue for personalised healthcare through noninvasive, patient-friendly, and POCT options (**Figure 1**). The future of vaginal microbiome health in clinical practice will leverage emerging technologies for more precise and personalised diagnosis and treatment. As understanding of the vaginal microbiome deepens, treatments tailored to an individual’s unique microbiome profile provide a potential avenue for the development of innovative treatments. Emerging evidence suggests that specific bacterial species may be associated with distinct disease states. However, the lack of standardisation currently limits our ability to reliably identify consistent microbial associations across studies. To advance the diagnostic potential of vaginal microbiome profiling, it is crucial to conduct further research focusing on standardised and optimised protocols to improve data comparability, enhance reproducibility, and support the development of a robust, disease-specific microbial biomarker database. By accurately analysing the composition and diversity of the vaginal microbiome, these diagnostics can enable early detection of conditions such as BV, STIs, and more complex issues such as pre-term birth, infertility and gynaecological cancers. There is a critical need to accelerate the development of improved techniques for providing accurate and early diagnoses of conditions affecting women’s health. The implementation of vaginal microbiome diagnostics into clinical practice holds the potential to transform women’s health, providing more precise, early, effective, and personalised care.

## References

[B1] Al-Memar M. Bobdiwala S. Fourie H. Mannino R. Lee Y. S. Smith A.. (2020). The association between vaginal bacterial composition and miscarriage: a nested case–control study. BJOG. doi: 10.1111/1471-0528.15972 PMC6972675 31573753

[B2] Amsel R. Totten P. A. Spiegel C. A. Chen K. C.S. Eschenbach D. Holmes K. K. (1983). Nonspecific vaginitis. Diagnostic criteria and microbial and epidemiologic associations. Am. J. Med. doi: 10.1016/0002-9343(83)91112-9 6600371

[B3] Anwar S. Mate R. Sinnakandu P. Hassall L. Tierney S. Vinci C.. (2023). EXPERT COMMITTEE ON BIOLOGICAL STANDARDIZATION Geneva, 16 to 19 October 2023 A WHO collaborative study to evaluate the candidate WHO International Reference Reagent for DNA extraction of the Gut Microbiome. Available at: www.who.int.

[B4] Aquino C. I. Nicosia A. Ligori A. Volpicelli A. I. Surico D. (2024). Microbiota status and endometrial cancer: A narrative review about possible correlations in affected versus healthy patients. Sci 6. doi: 10.3390/sci6040075

[B5] Ata B. Yildiz S. Turkgeldi E. Brocal V. P. Dinleyici E. C. Moya A.. (2019). The endobiota study: comparison of vaginal, cervical and gut microbiota between women with stage 3/4 endometriosis and healthy controls. Sci. Rep. doi: 10.1038/s41598-019-39700-6 PMC6379373 30778155

[B6] Bacterial vaginosis. Available online at: https://www.who.int/news-room/fact-sheets/detail/bacterial-vaginosis (Accessed January 28, 2025).

[B7] Baud A. Hillion K. H. Plainvert C. Tessier V. Tazi A. Mandelbrot L.. (2023). Microbial diversity in the vaginal microbiota and its link to pregnancy outcomes. Sci. Rep. doi: 10.1038/s41598-023-36126-z PMC10239749 37271782

[B8] Bayar E. MacIntyre D. A. Sykes L. Mountain K. Parks T. P. Lee P. P.. (2023). Safety, tolerability, and acceptability of Lactobacillus crispatus CTV-05 (LACTIN-V) in pregnant women at high-risk of preterm birth. Benef Microbes. doi: 10.3920/BM2022.0084 36815494

[B9] Bennett P. R. Brown R. G. MacIntyre D. A. (2020). Vaginal microbiome in preterm rupture of membranes. Obstet. Gynecol. Clin. North Am. doi: 10.1016/j.ogc.2020.08.001 33121642

[B10] Brown R. G. Marchesi J. R. Lee Y. S. Smith A. Lehne B. Kindinger L. M.. (2018). Vaginal dysbiosis increases risk of preterm fetal membrane rupture, neonatal sepsis and is exacerbated by erythromycin. BMC Med. doi: 10.1186/s12916-017-0999-x PMC5782380 29361936

[B11] Brown R. G. Al-Memar M. Marchesi J. R. Lee Y. S. Smith A. Chan D.. (2019). Establishment of vaginal microbiota composition in early pregnancy and its association with subsequent preterm prelabor rupture of the fetal membranes. Trans. Res. doi: 10.1016/j.trsl.2018.12.005 PMC6489901 30633889

[B12] Brusselaers N. Shrestha S. van de Wijgert J. Verstraelen H. (2019). Vaginal dysbiosis and the risk of human papillomavirus and cervical cancer: systematic review and meta-analysis. Am. J. Obstet. Gynecol. doi: 10.1016/j.ajog.2018.12.011 30550767

[B13] Budding A. E. Grasman M. E. Lin F. Bogaards J. A. Soeltan‐Kaersenhout D. J. Vandenbroucke‐Grauls C. M. J. E.. (2010). IS-pro: high-throughput molecular fingerprinting of the intestinal microbiota. FASEB J. doi: 10.1096/fj.10-156190 20643909

[B14] Cartwright C. P. Lembke B. D. Ramachandran K. Body B. A. Nye M. B. Rivers C. A.. (2012). Development and validation of a semiquantitative, multitarget PCR assay for diagnosis of bacterial vaginosis. J. Clin. Microbiol. doi: 10.1128/JCM.00506-12 PMC3405607 22535982

[B15] Caruso G. Giammanco A. Virruso R. Fasciana T. (2021). Current and future trends in the laboratory diagnosis of sexually transmitted infections. Int. J. Environ. Res. Public Health. doi: 10.3390/ijerph18031038 PMC7908473 33503917

[B16] Casas C. P. R. Albuquerque R. de C. R. de Loureiro R. B. Gollner A. M. Freitas M. G. de Duque G. P. do N.. (2022). Cervical cancer screening in low- and middle-income countries: A systematic review of economic evaluation studies. Clinics. doi: 10.1016/j.clinsp.2022.100080 PMC9335392 35905574

[B17] Ceccarani C. Foschi C. Parolin C. D’Antuono A. Gaspari V. Consolandi C.. (2019). Diversity of vaginal microbiome and metabolome during genital infections. Sci. Rep. 9, 1–12. doi: 10.1038/s41598-019-50410-x 31575935 PMC6773718

[B18] Cervical cancer. (n.d.). Available online at: https://www.who.int/news-room/fact-sheets/detail/cervical-cancer (Accessed January 28, 2025).

[B19] Chee W. J. Y. Chew S. Y. Than L. T. L. (2020). Vaginal microbiota and the potential of Lactobacillus derivatives in maintaining vaginal health. Microb. Cell Fact 19, 1–24. doi: 10.1186/s12934-020-01464-4 33160356 PMC7648308

[B20] Chen X. Lu Y. Chen T. Li R. (2021). The female vaginal microbiome in health and bacterial vaginosis. Front. Cell Infect. Microbiol. 11, 1–15. doi: 10.3389/fcimb.2021.631972 PMC8058480 33898328

[B21] Choi S. Y. Choi J. H. (2024). Ovarian cancer and the microbiome: connecting the dots for early diagnosis and therapeutic innovations—A review. Medicina (Lithuania) 60. doi: 10.3390/medicina60030516 PMC10972291 38541242

[B22] Chu C. M. Lowder J. L. (2018). Diagnosis and treatment of urinary tract infections across age groups. Am. J. Obstet. Gynecol. doi: 10.1016/j.ajog.2017.12.231 29305250

[B23] d’Humières C. Salmona M. Dellière S. Leo S. Rodriguez C. Angebault C.. (2021). The potential role of clinical metagenomics in infectious diseases: therapeutic perspectives. Drugs 81, 1453–1466. doi: 10.1007/s40265-021-01572-4 34328626 PMC8323086

[B24] Da Silva A. S. Baines G. Araklitis G. Robinson D. Cardozo L. (2021). Modern management of genitourinary syndrome of menopause. Fac Rev. 10. doi: 10.12703/r/10-25 PMC7946389 33718942

[B25] Department of Health (2016). Safer maternity care 1–24. Available at: https://www.gov.uk/government/publications/safer-maternity-care

[B26] d’Humières C. Salmona M. Dellière S. Leo S. Rodriguez C. Angebault C.. (2021). The potential role of clinical metagenomics in infectious diseases: therapeutic perspectives. Drugs 81, 1453–1466. doi: 10.1007/s40265-021-01572-4 34328626 PMC8323086

[B27] EMMA Endometrial microbiome metagenomic analysis. (n.d.). Available online at: https://www.igenomix.co.uk/genetic-solutions/emma-clinics/ (Accessed January 28, 2025).

[B28] Endometrial Health EMMA and ALICE tests. Care Fertility (n.d.). Available at: https://carefertility.com/treatments/emma-and-alice (Accessed January 28, 2025).

[B29] Eschenbach D. A. (1993). Bacterial vaginosis and anaerobes in obstetric-gynecologic infection. Clin. Infect. Dis. doi: 10.1093/clinids/16.Supplement_4.S282 8324132

[B30] Ferreira C. S. T. Da Silva M. G. De Pontes L. G. Dos Santos L. D. Marconi C. (2018). Protein content of cervicovaginal fluid is altered during bacterial vaginosis. J. Low Genit Tract Dis. doi: 10.1097/LGT.0000000000000367 29474232

[B31] Gao X. S. Laven J. Louwers Y. Budding A. Schoenmakers S. (2022). Microbiome as a predictor of implantation. Curr. Opin. Obstetrics Gynecology. doi: 10.1097/GCO.0000000000000782 35645010

[B32] García-Velasco J. A. Budding D. Campe H. Malfertheiner S. F. Hamamah S. Santjohanser C.. (2020). The reproductive microbiome – clinical practice recommendations for fertility specialists. Reprod. BioMedicine Online. doi: 10.1016/j.rbmo.2020.06.014 32753361

[B33] Geller S. E. Koch A. Pellettieri B. Carnes M. (2011). Inclusion, analysis, and reporting of sex and race/ethnicity in clinical trials: Have we made progress? J. Womens Health 20, 315–320. doi: 10.1089/jwh.2010.2469 PMC3058895 21351877

[B34] Gliniewicz K. Schneider G. M. Ridenhour B. J. Williams C. J. Song Y. Farage M. A.. (2019). Comparison of the vaginal microbiomes of premenopausal and postmenopausal women. Front. Microbiol. doi: 10.3389/fmicb.2019.00193 PMC6382698 30837959

[B35] Gosmann C. Anahtar M. N. Handley S. A. Farcasanu M. Abu-Ali G. Bowman B. A.. (2017). Lactobacillus-deficient cervicovaginal bacterial communities are associated with increased HIV acquisition in young South African women. Immunity. doi: 10.1016/j.immuni.2016.12.013 PMC5270628 28087240

[B36] Gudnadottir U. Debelius J. W. Du J. Hugerth L. W. Danielsson H. Schuppe-Koistinen I.. (2022). The vaginal microbiome and the risk of preterm birth: a systematic review and network meta-analysis. Sci. Rep. doi: 10.1038/s41598-022-12007-9 PMC9106729 35562576

[B37] Han Y. Liu Z. Chen T. (2021). Role of vaginal microbiota dysbiosis in gynecological diseases and the potential interventions. Front. Microbiol. doi: 10.3389/fmicb.2021.643422 PMC8249587 34220737

[B38] Harding C. Clavica F. Averbeck M. A. Da Silva A. Drake M. J. Gajewski J. B.. (2024). Can we prevent recurrent UTIs without antibiotics, in both those who do and do not use catheters? ICI-RS 2024. Neurourol Urodyn. 1–7. doi: 10.1002/nau.25641 39718154

[B39] Hassall J. Coxon C. Patel V. C. Goldenberg S. D. Sergaki C. (2024). Limitations of current techniques in clinical antimicrobial resistance diagnosis: examples and future prospects. NPJ Antimicrobials Resistance 2, 1–8. doi: 10.1038/s44259-024-00033-8 39843577 PMC11721362

[B40] Hickey R. J. Zhou X. Pierson J. D. Ravel J. Forney L. J. (2012). Understanding vaginal microbiome complexity from an ecological perspective. Trans. Res. 160, 267–282. doi: 10.1016/j.trsl.2012.02.008 PMC3444549 22683415

[B41] Holliday M. Uddipto K. Castillo G. Vera L. E. Quinlivan J. A. Mendz G. L. (2023). Insights into the genital microbiota of women who experienced fetal death in utero. Microorganisms. doi: 10.3390/microorganisms11081877 PMC10456767 37630436

[B42] Hong X. Qin P. Huang K. Ding X. Ma J. Xuan Y.. (2020). Association between polycystic ovary syndrome and the vaginal microbiome: A case-control study. Clin. Endocrinol. (Oxf). doi: 10.1111/cen.14198 32311120

[B43] Huang C. Gin C. Fettweis J. Foxman B. Gelaye B. MacIntyre D. A.. (2023). Meta-analysis reveals the vaginal microbiome is a better predictor of earlier than later preterm birth. BMC Biol. doi: 10.1186/s12915-023-01702-2 PMC10518966 37743497

[B44] Huang B. Fettweis J. M. Brooks J. P. Jefferson K. K. Buck G. A. (2014). The changing landscape of the vaginal microbiome. Clinics Lab. Med. doi: 10.1016/j.cll.2014.08.006 PMC4254509 25439274

[B45] Invivo Healthcare The human microbiome company. Available online at: https://invivohealthcare.com/?gad_source=1&gbraid=0AAAAA9yfhAiyCBJlxG7brj8RtQngUcJk_&gclid=EAIaIQobChMI9P_LypqCigMVbZhQBh3NNCimEAAYAiAAEgIt0vD_BwE (Accessed January 28, 2025).

[B46] Ishimwe J. A. (2021). Maternal microbiome in preeclampsia pathophysiology and implications on offspring health. Physiol. Rep. doi: 10.14814/phy2.14875 PMC8157769 34042284

[B47] It’s about time to focus on women’s health. (2023). Nat. Rev. Bioengineering 1, 379–379. doi: 10.1038/s44222-023-00081-1

[B48] Jacobson D. Moore K. Gunderson C. Rowland M. Austin R. Prasad T.. (2021). Shifts in gut and vaginal microbiomes are associated with cancer recurrence time in women with ovarian cancer. PeerJ. doi: 10.7717/peerj.11574 PMC8214851 34178459

[B49] Jepsen I. E. Saxtorph M. H. Englund A. L. M. Petersen K. B. Wissing M. L. M. Hviid T. V. F.. (2022). Probiotic treatment with specific lactobacilli does not improve an unfavorable vaginal microbiota prior to fertility treatment—A randomized, double-blinded, placebo-controlled trial. Front. Endocrinol. (Lausanne). doi: 10.3389/fendo.2022.1057022 PMC9751370 36531460

[B50] Kashyap P. C. Chia N. Nelson H. Segal E. Elinav E. (2017). Microbiome at the frontier of personalized medicine. Mayo Clin. Proc. 92, 1855–1864. doi: 10.1016/j.mayocp.2017.10.004 29202942 PMC5730337

[B51] Khan K. N. Kitajima M. Hiraki K. Yamaguchi N. Katamine S. Matsuyama T.. (2010). Escherichia coli contamination of menstrual blood and effect of bacterial endotoxin on endometriosis. Fertil Steril. doi: 10.1016/j.fertnstert.2010.04.053 20627244

[B52] Koedooder R. Singer M. Schoenmakers S. Savelkoul P. H. M. Morré S. A. De Jonge J. D.. (2019). The vaginal microbiome as a predictor for outcome of *in vitro* fertilization with or without intracytoplasmic sperm injection: A prospective study. Hum. Reprod. doi: 10.1093/humrep/dez065 31119299

[B53] Kunaseth J. Waiyaput W. Chanchaem P. Sawaswong V. Permpech R. Payungporn S.. (2022). Vaginal microbiome of women with adenomyosis: A case-control study. PloS One. doi: 10.1371/journal.pone.0263283 PMC8849446 35171931

[B54] Lamont R. F. Sawant S. R. (2005). Infection in the prediction and antibiotics in the prevention of spontaneous preterm labour and preterm birth. Minerva Ginecologica. doi: 10.1046/j.1471-0528.2003.00034.x 16170287

[B55] Ley R. E. (2016). Gut microbiota in 2015: Prevotella in the gut: Choose carefully. Nat. Rev. Gastroenterol. Hepatol. doi: 10.1038/nrgastro.2016.4 26828918

[B56] Li C. Gu Y. He Q. Huang J. Song Y. Wan X.. (2021). Integrated analysis of microbiome and transcriptome data reveals the interplay between commensal bacteria and fibrin degradation in endometrial cancer. Front. Cell Infect. Microbiol. doi: 10.3389/fcimb.2021.748558 PMC8490766 34621695

[B57] McClelland R. S. Lingappa J. R. Srinivasan S. Kinuthia J. John-Stewart G. C. Jaoko W.. (2018). Evaluation of the association between the concentrations of key vaginal bacteria and the increased risk of HIV acquisition in African women from five cohorts: a nested case-control study. Lancet Infect. Dis. doi: 10.1016/S1473-3099(18)30058-6 PMC6445552 29396006

[B58] McCormack D. Koons K. (2019). Sexually transmitted infections. Emerg Med Clin North Am. doi: 10.1016/j.emc.2019.07.009 31563204

[B59] Merone L. Tsey K. Russell D. Nagle C. (2022). Sex inequalities in medical research: A systematic scoping review of the literature. Women’s Health Rep. 3, 49–59. doi: 10.1089/whr.2021.0083 PMC8812498 35136877

[B60] Mitra A. MacIntyre D. A. Lee Y. S. Smith A. Marchesi J. R. Lehne B.. (2015). Cervical intraepithelial neoplasia disease progression is associated with increased vaginal microbiome diversity. Sci. Rep. doi: 10.1038/srep16865 PMC4648063 26574055

[B61] Mitra A. MacIntyre D. A. Ntritsos G. Smith A. Tsilidis K. K. Marchesi J. R.. (2020). The vaginal microbiota associates with the regression of untreated cervical intraepithelial neoplasia 2 lesions. Nat. Commun. doi: 10.1038/s41467-020-15856-y PMC7181700 32332850

[B62] Muzny C. A. Blanchard E. Taylor C. M. Aaron K. J. Talluri R. Griswold M. E.. (2018). Identification of key bacteria involved in the induction of incident bacterial vaginosis: A prospective study. J. Infect. Dis. doi: 10.1093/infdis/jiy243 PMC6093354 29718358

[B63] Nugent R. P. Krohn M. A. Hillier S. L. (1991). Reliability of diagnosing bacterial vaginosis is improved by a standardized method of gram stain interpretation. J. Clin. Microbiol. doi: 10.1128/jcm.29.2.297-301.1991 PMC269757 1706728

[B64] Okunade K. S. (2020). Human papillomavirus and cervical cancer. J. Obstetrics Gynaecology (Lahore). doi: 10.1080/01443615.2019.1634030 PMC7062568 31500479

[B65] Ovarian cancer statistics World cancer research fund (n.d.). Available online at: https://www.wcrf.org/preventing-cancer/cancer-statistics/ovarian-cancer-statistics/ (Accessed January 28, 2025).

[B66] Pan Z. Dai J. Zhang P. Ren Q. Wang X. Yan S.. (2024). Vaginal microbiome differences between patients with adenomyosis with different menstrual cycles and healthy controls. BMC Microbiol. 24, 1–14. doi: 10.1186/s12866-024-03339-9 39068412 PMC11282752

[B67] Park S. Oh D. Heo H. Lee G. Kim S. M. Ansari A. Z.. (2021). Prediction of preterm birth based on machine learning using bacterial risk score in cervicovaginal fluid. Am. J. Reprod. Immunol. doi: 10.1111/aji.13435 33905152

[B68] Park S. Moon J. Kang N. Kim Y. H. You Y. A. Kwon E.. (2022). Predicting preterm birth through vaginal microbiota, cervical length, and WBC using a machine learning model. Front. Microbiol. doi: 10.3389/fmicb.2022.912853 PMC9378785 35983325

[B69] Peebles K. Velloza J. Balkus J. E. McClelland R. S. Barnabas R. V. (2019). High global burden and costs of bacterial vaginosis: A systematic review and meta-analysis. Sexually Transmitted Dis. doi: 10.1097/OLQ.0000000000000972 30624309

[B70] Peelen M. J. Luef B. M. Lamont R. F. de Milliano I. Jensen J. S. Limpens J.. (2019). The influence of the vaginal microbiota on preterm birth: A systematic review and recommendations for a minimum dataset for future research. Placenta. doi: 10.1016/j.placenta.2019.03.011 31047708

[B71] Peipert J. F. Lapane K. L. Allsworth J. E. Redding C. A. Blume J. D. Stein M. D. (2008). Bacterial vaginosis, race, and sexually transmitted infections: Does race modify the association? Sex Transm Dis. doi: 10.1097/OLQ.0b013e31815e4179 18360319

[B72] Pereira M. P. Jones S. Costin J. M. (2024). Association of polycystic ovarian syndrome (PCOS) with vaginal microbiome dysbiosis: A scoping review. Cureus 16, 1–9. doi: 10.7759/cureus.62611 PMC11257764 39027755

[B73] Portman D. J. Gass M. L. S. Kingsberg S. Archer D. Bachmann G. Burrows L.. (2014). Genitourinary syndrome of menopause: New terminology for vulvovaginal atrophy from the international society for the study of women’s sexual health and the North American Menopause Society. Menopause. doi: 10.1097/gme.0000000000000329 25153131

[B74] Pruski P. Correia G. D. S. Lewis H. V. Capuccini K. Inglese P. Chan D.. (2021). Direct on-swab metabolic profiling of vaginal microbiome host interactions during pregnancy and preterm birth. Nat. Commun. doi: 10.1038/s41467-021-26215-w PMC8514602 34645809

[B75] Ratiner K. Ciocan D. Abdeen S. K. Elinav E. (2024). Utilization of the microbiome in personalized medicine. Nat. Rev. Microbiol. 22, 291–308. doi: 10.1038/s41579-023-00998-9 38110694

[B76] Ravel J. Gajer P. Abdo Z. Schneider G. M. Koenig S. S. K. McCulle S. L.. (2011). Vaginal microbiome of reproductive-age women. Proc. Natl. Acad. Sci. U.S.A. 108, 4680–4687. doi: 10.1073/pnas.1002611107 20534435 PMC3063603

[B77] Richard M. L. Liguori G. Lamas B. Brandi G. da Costa G. Hoffmann T. W.. (2018). Mucosa-associated microbiota dysbiosis in colitis associated cancer. Gut Microbes. doi: 10.1080/19490976.2017.1379637 PMC5989788 28914591

[B78] Saadaoui M. Singh P. Ortashi O. Al Khodor S. (2023). Role of the vaginal microbiome in miscarriage: exploring the relationship. Front. Cell. Infect. Microbiol. doi: 10.3389/fcimb.2023.1232825 PMC10533927 37780845

[B79] Sekaran K. Varghese R. P. Gopikrishnan M. Alsamman A. M. El Allali A. Zayed H.. (2023). Unraveling the dysbiosis of vaginal microbiome to understand cervical cancer disease etiology—An explainable AI approach. Genes (Basel). doi: 10.3390/genes14040936 PMC10137380 37107694

[B80] Ser H. L. Au Yong S. J. Shafiee M. N. Mokhtar N. M. Ali R. A. R. (2023). Current updates on the role of microbiome in endometriosis: A narrative review. Microorganisms. doi: 10.3390/microorganisms11020360 PMC9962481 36838325

[B81] Sergaki C. Anwar S. Hassall J. Logan A. Rigsby P. Rijpkema S.. (2022). EXPERT COMMITTEE ON BIOLOGICAL STANDARDIZATION Geneva, 4 to 8 April 2022 A WHO collaborative study to evaluate the candidate 1 st WHO International Reference Reagents for Gut Microbiome analysis by Next-Generation Sequencing National Institute for Biolog 1–63.

[B82] Sexually transmitted infections (STIs). Available online at: https://www.who.int/news-room/fact-sheets/detail/sexually-transmitted-infections-(stis) (Accessed January 28, 2025).

[B83] Shahid M. Quinlivan J. A. Peek M. Castaño-Rodríguez N. Mendz G. L. (2022). Is there an association between the vaginal microbiome and first trimester miscarriage? A prospective observational study. J. Obstetrics Gynaecology Res. doi: 10.1111/jog.15086 34761471

[B84] Sharifian K. Shoja Z. Jalilvand S. (2023). The interplay between human papillomavirus and vaginal microbiota in cervical cancer development. Virol. J. doi: 10.1186/s12985-023-02037-8 PMC10114331 37076931

[B85] Sola-Leyva A. Pérez-Prieto I. Molina N. M. Vargas E. Ruiz-Durán S. Leonés-Baños I.. (2023). Microbial composition across body sites in polycystic ovary syndrome: a systematic review and meta-analysis. Reprod. BioMedicine Online. doi: 10.1016/j.rbmo.2023.03.016 37208218

[B86] Srinivasan S. Hoffman N. G. Morgan M. T. Matsen F. A. Fiedler T. L. Hall R. W.. (2012). Bacterial communities in women with bacterial vaginosis: High resolution phylogenetic analyses reveal relationships of microbiota to clinical criteria. PloS One. doi: 10.1371/journal.pone.0037818 PMC3377712 22719852

[B87] Sung H. Ferlay J. Siegel R. L. Laversanne M. Soerjomataram I. Jemal A.. (2021). Global cancer statistics 2020: GLOBOCAN estimates of incidence and mortality worldwide for 36 cancers in 185 countries. CA Cancer J. Clin. doi: 10.3322/caac.21660 33538338

[B88] Taithongchai A. Reid F. Agro E. F. Rosato E. Bianchi D. Serati M.. (2024). Are we able to optimize outcomes and predict complications in pelvic floor surgery with a better understanding of hormonal, microbial and other factors? A report from the ICI-RS 2024. Neurourol Urodyn, 1–8. doi: 10.1002/nau.25645 39704249

[B89] The CA125 blood test for ovarian cancer has been re-evaluated (n.d.). Available online at: https://news.cancerresearchuk.org/2020/10/28/an-existing-blood-test-for-ovarian-cancer-has-been-re-evaluated/ (Accessed January 28, 2025).

[B90] Thomson A. J. (2019). Care of women presenting with suspected preterm prelabour rupture of membranes from 24 + 0 weeks of gestation: green-top guideline no. 73. BJOG. doi: 10.1111/1471-0528.15803 31207667

[B91] Van De Wijgert J. H. H. M. Morrison C. S. Brown J. Kwok C. Van Der Pol B. Chipato T.. (2009). Disentangling contributions of reproductive tract infections to hiv acquisition in african women. Sex Transm Dis. doi: 10.1097/OLQ.0b013e3181a4f695 19434010

[B92] Vitale S. G. Ferrari F. Ciebiera M. Zgliczyńska M. Rapisarda A. M. C. Vecchio G. M.. (2022). The role of genital tract microbiome in fertility: A systematic review. Int. J. Mol. Sci. doi: 10.3390/ijms23010180 PMC8745627 35008605

[B93] Wang L. Koppolu S. Chappell C. Moncla B. J. Hillier S. L. Mahal L. K. (2015). Studying the effects of reproductive hormones and bacterial vaginosis on the glycome of lavage samples from the cervicovaginal cavity. PloS One. doi: 10.1371/journal.pone.0127021 PMC4439148 25993513

[B94] Wang Y. Thakur R. Shen Q. He Y. Chen C. (2023). Influences of vaginal microbiota on human papillomavirus infection and host immune regulation: What we have learned? Decoding Infection Transm. doi: 10.1016/j.dcit.2023.07.001

[B95] Wei B. Chen Y. Lu T. Cao W. Tang Z. Yang H. (2022). Correlation between vaginal microbiota and different progression stages of cervical cancer. Genet. Mol. Biol. doi: 10.1590/1678-4685-gmb-2020-0450 PMC8967114 35320337

[B96] Wessels J. M. Domínguez M. A. Leyland N. A. Agarwal S. K. Foster W. G. (2021). Endometrial microbiota is more diverse in people with endometriosis than symptomatic controls. Sci. Rep. doi: 10.1038/s41598-021-98380-3 PMC8460742 34556738

[B97] White B. A. Creedon D. J. Nelson K. E. Wilson B. A. (2011). The vaginal microbiome in health and disease. Trends Endocrinol. Metab. doi: 10.1016/j.tem.2011.06.001 PMC3183339 21757370

[B98] Witkin S. S. Linhares I. M. (2017). Why do lactobacilli dominate the human vaginal microbiota? BJOG 124, 606–611. doi: 10.1111/1471-0528.14390 28224747

[B99] World Health Organization (2021). Infertility prevalence estimates. Geneva, Switzerland: WHO Press, World Health Organization.

[B100] Yeoman C. J. Thomas S. M. Miller M. E. B. Ulanov A. V. Torralba M. Lucas S.. (2013). A multi-omic systems-based approach reveals metabolic markers of bacterial vaginosis and insight into the disease. PloS One. doi: 10.1371/journal.pone.0056111 PMC3566083 23405259

[B101] Yim E.-K. Park J.-S. (2005). The role of HPV E6 and E7 oncoproteins in HPV-associated cervical carcinogenesis. Cancer Res. Treat. doi: 10.4143/crt.2005.37.6.319 PMC2785934 19956366

